# Genome-wide identification, characteristics and expression of the prolamin genes in *Thinopyrum elongatum*

**DOI:** 10.1186/s12864-021-08088-x

**Published:** 2021-12-02

**Authors:** Wenyang Ge, Yu Gao, Shoushen Xu, Xin Ma, Hongwei Wang, Lingrang Kong, Silong Sun

**Affiliations:** State Key Laboratory of Crop Biology, College of Agronomy, Shandong Agricultural University, Tai’an, 271018 China

**Keywords:** *Thinopyrum elongatum*, Prolamins, Expression, Evolution, Celiac disease

## Abstract

**Background:**

Prolamins, unique to Gramineae (grasses), play a key role in the human diet. *Thinopyrum elongatum* (syn. *Agropyron elongatum* or *Lophopyrum elongatum*), a grass of the Triticeae family with a diploid E genome (2n = 2x = 14), is genetically well-characterized, but little is known about its prolamin genes and the relationships with homologous loci in the Triticeae species*.*

**Results:**

In this study, a total of 19 α-gliadin, 9 γ-gliadin, 19 ω-gliadin, 2 high-molecular-weight glutenin subunit (HMW-GS), and 5 low-molecular-weight glutenin subunit (LMW-GS) genes were identified in the *Th. elongatum* genome. Micro-synteny and phylogenetic analysis revealed dynamic changes of prolamin gene regions and genetic affinities among *Th. elongatum*, *Triticum aestivum*, *T. urartu* and *Aegilops tauschii*. The *Th. elongatum* genome, like the B subgenome of *T. aestivum*, only contained celiac disease epitope DQ8-glia-α1/DQ8.5-glia-α1, which provided a theoretical basis for the low gluten toxicity wheat breeding. The transcriptome data of *Th. elongatum* exhibited differential expression in quantity and pattern in the same subfamily or different subfamilies. Dough rheological properties of *T. aestivum*-*Th. elongatum* disomic substitution (DS) line 1E(1D) showed higher peak height values than that of their parents, and DS6E(6D) exhibited fewer α-gliadins, which indicates the potential usage for wheat quality breeding.

**Conclusions:**

Overall, this study provided a comprehensive overview of the prolamin gene family in *Th. elongatum*, and suggested a promising use of this species in the generation of improved wheat breeds intended for the human diet.

**Supplementary Information:**

The online version contains supplementary material available at 10.1186/s12864-021-08088-x.

## Background

Prolamins (glutenins and gliadins), comprising 80% of wheat endosperm protein, are the main component of glutens [1]. High- and low-molecular-weight glutenin subunits (HMW-GSs and LMW-GSs) form polymeric proteins by interchain disulfide bonds, imparting the elasticity of dough. Gliadins, divided into α-, β-, γ-, and ω-gliadins, are monomeric proteins of 30–78 kDa that determine the ductility and viscosity of dough [2–5]. These unique properties determine the quality of wheat flour in various technological processes and enable the manufacture of a wide range of products such as bread, pasta, noodles, cakes, and pastries [6, 7]. Prolamin genes were reported to be closely linked and mainly located on a few chromosomes in common wheat [8]. The HMW-GS genes are encoded by the *Glu-1* loci on the long arm of the first homologous group [9]. The γ-gliadin and ω-gliadin genes are encoded by the *Gli-1* loci closely linked to the *Glu-3* loci encoding LMW-GS genes on the short arm of the same chromosome, on which HMW-GS genes are located [10]. The α-gliadin genes are encoded by the *Gli-2* loci on the short arm of the sixth homologous group [11].

Prolamin genes belong to a large family. Comprehensive understanding of these genes is challenging, but essential for improving the end-use quality of wheat flour [7]. Generally, *T. urartu* (AA, 2n = 2x = 14) and *Ae. Tauschii* (DD, 2n = 2x = 14) are regarded as donors of the A and D genomes of common wheat [12]. *Th. elongatum*, closely related to wheat, has excellent performance in biotic and abiotic stress resistance [13]. At present, genome data of these Triticeae species have been published, but the relationship among their prolamin genes has not been well described, including gene features and molecular characteristics [13–16]. High pseudogene rate was associated with gliadin families and this was estimated to be 87% of 230 distinct α-gliadin gene sequences in several diploid wheat species [1]. Hence, it is necessary to understand the expression profile of these genes to clarify the mechanism of gene activation. However, current research is still insufficient, especially regarding species other than common wheat [17].

Gene clusters of a gene family are often prone to genetic variations in copy number, sequence polymorphism, and expression [18]. Comparisons of these homologous regions between different genomes of related species will provide insights into gene differentiation, as well as local rearrangements [19]. For example, a comparison of the *Gli-2* loci between *T. dicoccoides* Korn and *T. aestivum* cv. Chinese Spring (CS) showed a large sequence difference between the two A subgenomes and a conservative region between the two B subgenomes [20]. To date, a detailed analysis of other genomes (except for the A, B, and D genomes) on the interval of these loci has not been reported.

Gluten is the most important source of proteins in common wheat for human beings. Unfortunately, prolamins of gluten are also responsible for certain intolerances, among which celiac disease (CD) is one of the most common wheat-related disorders [21]. CD is a chronic intestinal immune-mediated bowel disease, which occurs in genetically susceptible people and is caused by the intake of gluten [22]. α-gliadins are the main substance causing CD [18]. A breeding effort had been proposed to develop wheat with reduced immunoreactive epitopes while retaining baking functions [23]. Prolamin genes from wild relatives of wheat were reported to have low gluten toxicity and could be used to improve wheat quality by distant hybridization [24].

In this study, prolamin genes were obtained and comparative analysis were carried out between *Th. elongatum* and three related species (*T. aestivum*, *T. urartu*, and *Ae. tauschii*) in terms of gene numbers, molecular characteristics, micro-synteny, phylogenetic relationship, CD epitope content and dynamic expression pattern. Mixogram tests of disomic substitution (DS) line 1E(1D) was also performed. The results presented will provide helpful information of prolamin genes in Triticeae and future application in wheat breeding.

## Results

### Identification of the prolamin genes in *Th. Elongatum* and error correction in the related species

A total of 19 α-gliadin, 9 γ-gliadin, 19 ω-gliadin, 2 HMW-GS, and 5 LMW-GS genes were identified in the *Th. elongatum* genome while no δ-gliadin genes were detected (Additional file 1: Table S1). Prolamin genes in the related species were also collected and reannotated. In addition, some annotation errors were found in related species. For example, there were no sequences for *AET6Gv20125500.1* and *AET6Gv20126100.1* in the existing CDS and protein files of *Ae. tauschii*. By checking their positions provided in that study, we found an incorrect coordinate layout (Additional file 1: Table S2) [15]. Several δ-gliadins were also identified in *T. aestivum* genome (Additional file 1: Table S4). All prolamin genes found in this study were manually checked and corrected according to their structure information (Additional file 1: Table S3 and Table S4) [8, 25–28].

### Characteristics and sequence analysis of the prolamin gene family

All prolamin genes of four species were first named based on abbreviations of species names and chromosomal locations. Then, the isoelectric point (pI), molecular weight (MW) and other characteristics of putative functional prolamin genes were calculated (Additional file 1: Table S4). No obvious differences were found between *Th. elongatum* and other three species except for *Tel_LMW_glutenin_1E_1*, a special prolamin gene that has the highest MW and the longest protein sequence (Additional file 1: Table S4).

**Fig. 1:**
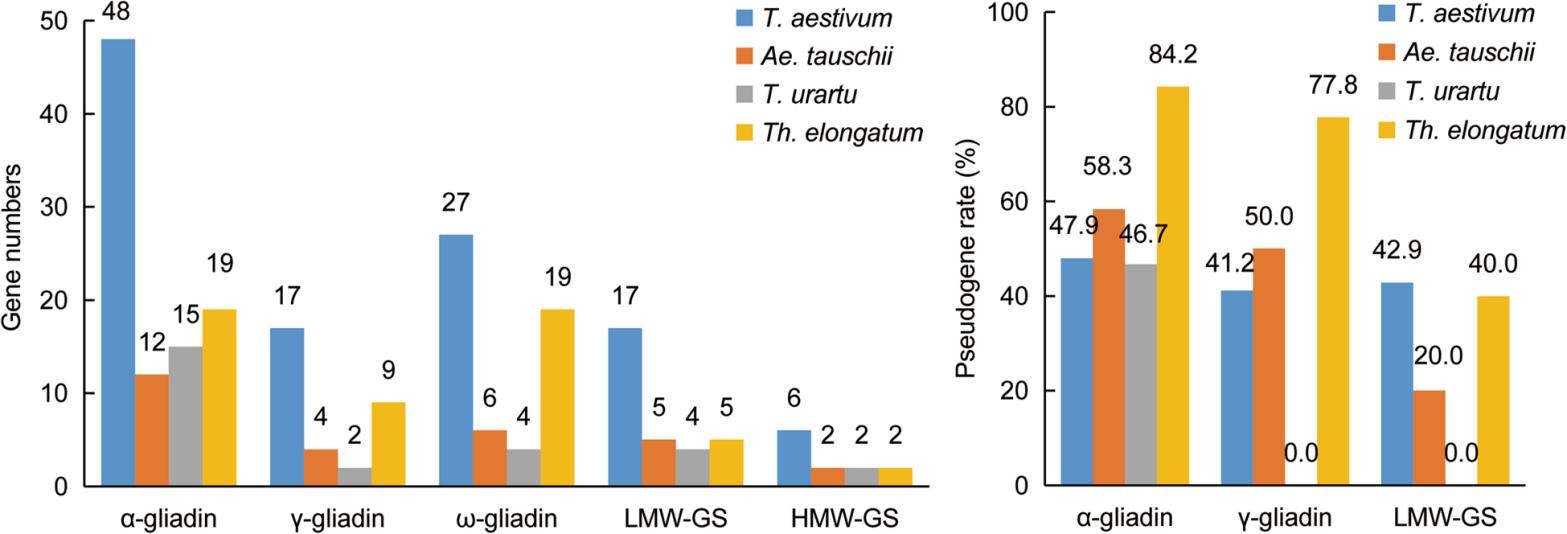
Characteristic comparisons of prolamin genes. **A** The number of each prolamin gene subfamily among *Th. elongatum*, *T. aestivum*, *T. urartu* and *Ae. tauschii*. **B** Pseudogene rates of α-gliadin, γ-gliadin and LMW-GS gene families among four selected species

The prolamin gene numbers showed variable patterns among different ploidy species (Fig. 1a). Only HMW-GS gene subfamily, carrying one x-type and one y-type in each diploid genome, follow a conservative pattern in the process of evolution [7]. In contrast, no specific pattern was observed among gliadin subfamilies across the species. What is particularly noticeable is that *Th. elongatum* had the highest numbers in three gliadin subfamilies of the three diploid species, and the number of ω-gliadin genes of *Th. elongatum* was about 3–5 times than that of the other two diploid species. A common feature of gliadin genes in the four species was that the numbers of α-gliadin genes were the largest, followed by ω-gliadin genes, and finally γ-gliadin genes.

The pseudogene rates of each prolamin subfamilies were calculated according to the pseudogene number based on the inference from coding sequences to protein sequences (Additional file 1: Table S3). *Th. elongatum* had the highest pseudogene rates in α-gliadin (84.2%) and γ-gliadin gene families (77.8%), while no pseudogenes were found in γ-gliadin and LMW-glutenin gene families in *T. urartu* (Fig. 1b).

### Chromosomal location and duplication of prolamin genes

**Fig. 2:**
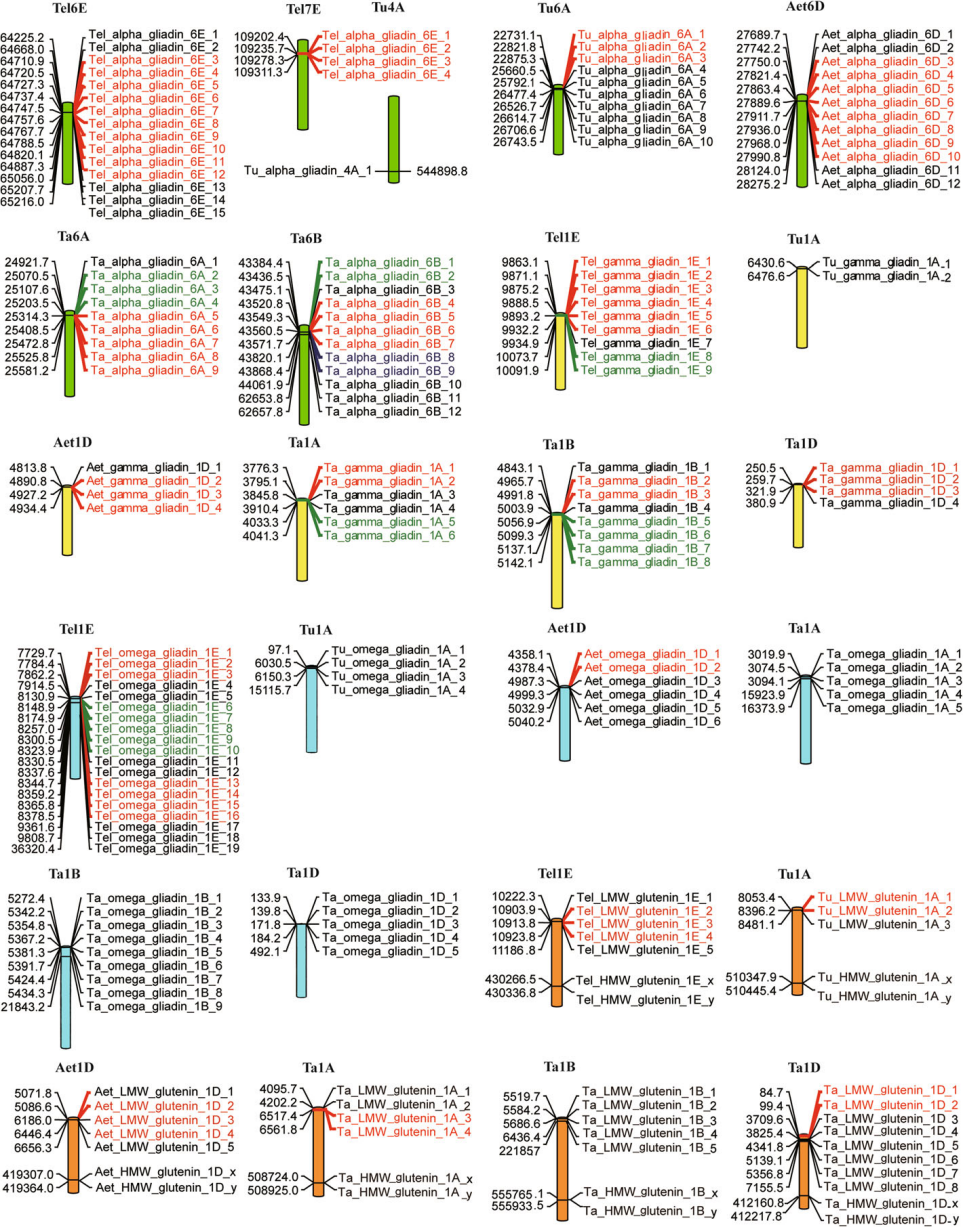
Chromosome location and gene duplication of prolamin genes. Tandem duplicated genes were marked by red, green or blue, respectively

Consistent with previous studies, the γ-gliadin, ω-gliadin and LMW-GS genes were distributed on the short arm, and the HMW-GS genes were located on the long arm of the first homologous group [10, 29, 30]. Most α-gliadins were mainly distributed on the sixth homologous group (Fig. 2) [11]. It is interesting to note that a new α-gliadin cluster with four pseudogenes was detected on the short arm of chromosome 7E (109,202,365-109,312,140 bp). Both tandem duplication and segmental duplication are associated with gene production. The proportions of tandem duplication genes of α-, γ-, ω-gliadins and LMW-GSs in *Th. elongatum* were 73.7, 88.9, 68.4 and 50%, which supports the hypothesis that the major expansion of prolamin genes was through tandem duplication.

**Fig. 3:**
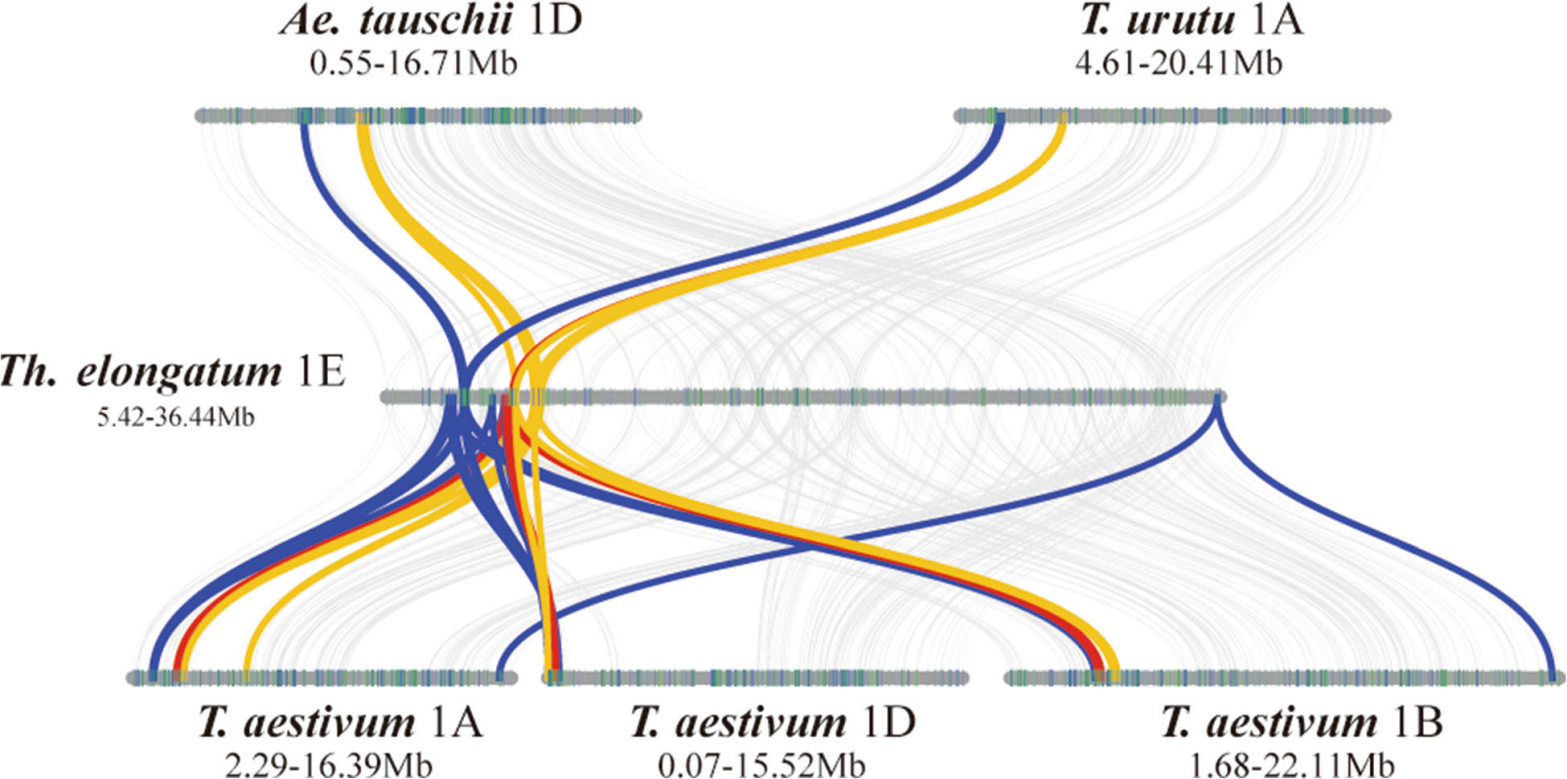
Micro-synteny analysis of ω-, γ-gliadin and LMW-GS genes*.* The relationship of these families is indicated by blue, red, and orange lines, respectively

To better understand the evolutionary mechanism of γ-gliadin, ω-gliadin and LMW-GS gene subfamilies, an interval covering the first to the last prolamin gene on the short arm of the first homologous group was used for micro-synteny analysis among the four genomes (Fig. 3). The results showed that all these regions were located in collinear blocks with an inversion occurred between *Glu-3* and *Gli-1* loci on chromosome 1D of common wheat and one isolated ω-gliadin gene existed in *Th. elongatum*, A and B subgenomes of common wheat but lost in *T. urartu, Ae. Tauschill* and the D subgenome. Micro-synteny analysis of α-gliadins in the four studied species exhibited multiple duplicates in the loci expect for D genomes (Additional file 2). Although gene collinearity appears to be retained, four of six genes including the two paralogous HMW-GS genes were reversed in the homologous region of the *T. urartu* genome (Additional file 2) [31]. Changes in these gene loci between related species provided evidences for the dynamic evolution of the prolamin gene family.

### Evolutionary analysis of prolamin subfamilies

Phylogenetic trees were constructed for each prolamin gene subfamilies to investigate the internal evolutionary relationship within Triticeae species. *GQ139526.1* (*Psathyrostachys huashanica*), *X13508.1* (*Hordeum vulgare*), *HQ293220.1* (*Dasypyrum villosum*) and *FJ481574.2* (*Eremopyrum triticeum*) were selected as outgroups, respectively.

**Fig. 4:**
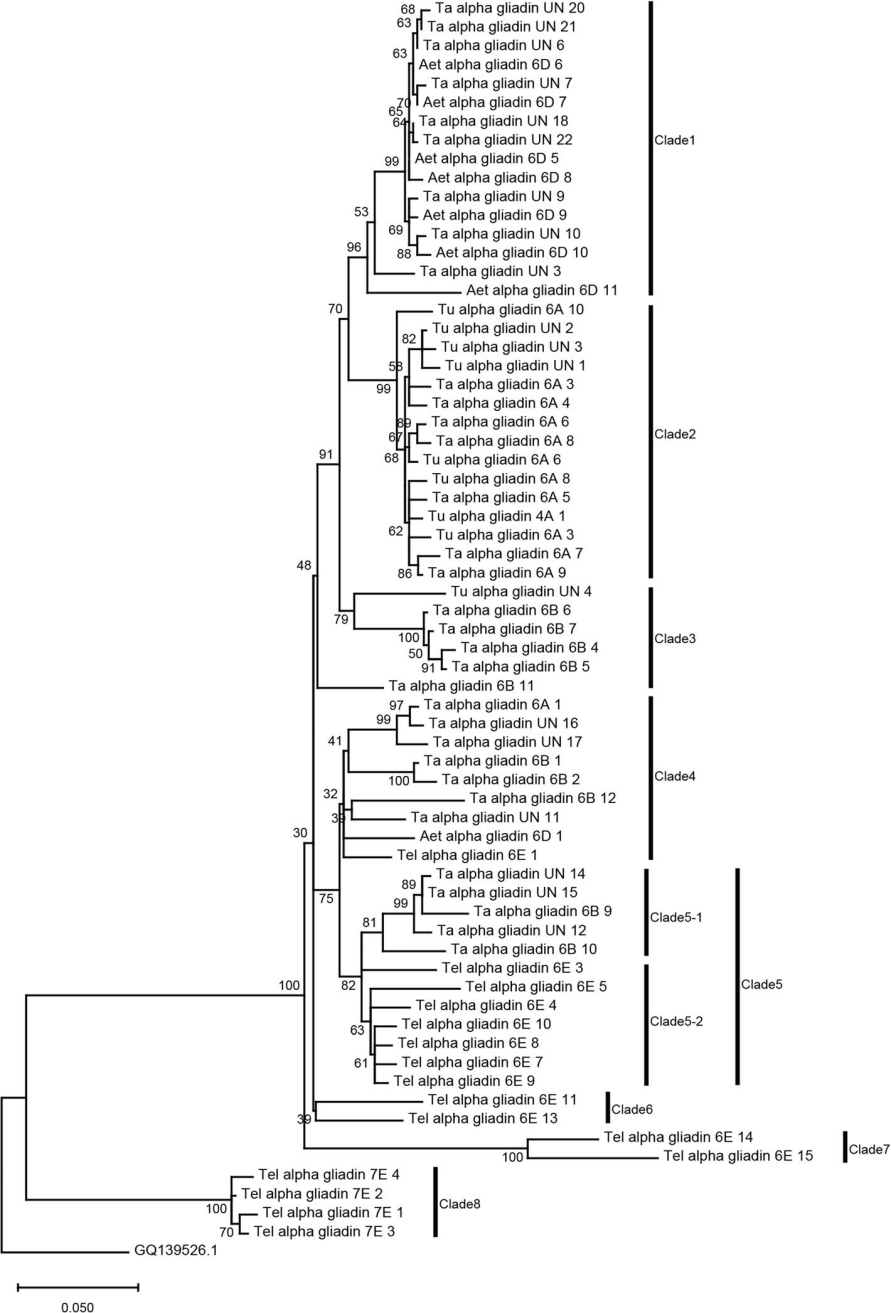
Phylogenetic tree of α-gliadin genes. The phylogenetic tree was constructed by MEGA X with the maximum likelihood (ML) method and 1000 bootstrap replications. GQ139528.1 was set as outgroup. Different clades are marked with vertical bars and the genes of different species can be judged by their names

All 66 α-gliadin genes were divided into 8 clades and genes from the same genome were generally clustered together (Fig. 4). It is interesting to note that 4 genes located on the 7E chromosome of *Th. elongatum* formed a separate clade (clade 8) with a high bootstrap value and had a closer genetic distance with the outgroup species, which indicates that these genes on chromosome 7E evolved earlier than those on chromosome 6E and formed as a result of different evolutionary trajectories in early stages. In HMW-GS gene subfamily, all 12 genes were well divided into 2 clades, representing y-type HMW-GSs (clade 1) and x-type HMW-GSs (clade 2) (Additional file 3) [14]. The topological structure of y-type HMW-GSs had some differences with x-type HMW-GSs, which might be related to the sequence variation of the C-terminal region of y-type HMW-GSs. The LMW-GS, γ-gliadin and ω-gliadin gene subfamilies showed similar phylogenetic relationship as α-gliadin and HMW-GS genes (Additional file 3 and Additional file 4).

### Distribution of CD epitopes in α-gliadins genes

The most influential T cell epitopes in CD patients are PFPQPQLPY (DQ2.5-glia-α1a), PYPQPQLPY (DQ2.5-glia-α1b), PQPQLPYPQ (DQ2.5-glia-α2), FRPQQQPYPQ (DQ2.5-glia-α3), and QGSFQPSQQ (DQ8-glia-α1/DQ8.5-glia-α1), as well as the most toxic 33-mer peptide (LQLQPFPQPQLPYPQPQLPYPQPQLPYPQPQPF) [32, 33]. About 93.3% α-gliadin genes contained DQ2.5-glia-α1a or DQ2.5-glia-α3 in the A genomes, but only 28.6% contained only one type epitope (DQ8-glia-α1/DQ8.5-glia-α1) in the B genomes (Additional file 1: Table S5). The epitope types in the D genome were the most abundant and all six types were identified. The E genome, like the B genome, also only contained one type epitope, DQ8-glia-α1/DQ8.5-glia-α1. 33-mer peptide was only detected in the D genome of common wheat, which was consisted with previous report [1]. DQ2.5-glia-α1b and DQ2.5-glia-α2 were also only found in the D genome and often existed in the form of multiple peptides, especially DQ2.5-glia-α2, and in contrast the other small peptides existed in the form of single peptides.

### Expression pattern of prolamin genes in *Th. Elongatum*

**Fig. 5:**
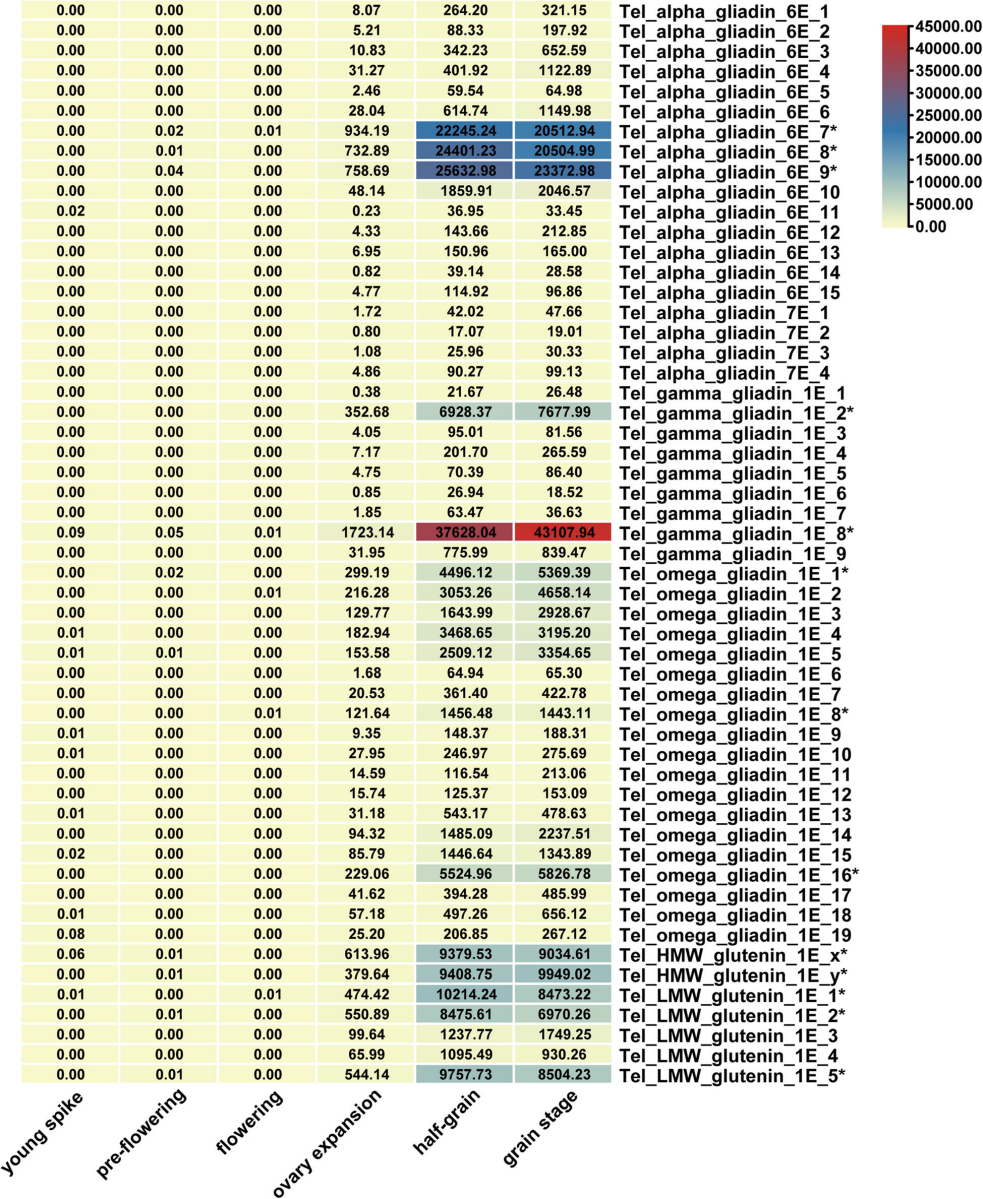
Expression profiles of prolamin genes. The putative functional genes were marked with an asterisk after the gene name

The expression of prolamin genes in *Th. elongatum* were investigated with transcriptome data collected at six development stages (Fig. 5 and Additional file 1: Table S6). Genes with predicted putative functions (Marked with asterisk) were tended to have a higher TPM value than pseudogenes, which indicted high accurate prediction of our study. Comparative analysis revealed that the expression pattern of prolamin genes in *Th. elongatum* were different in the same subfamily, which was one of the factors influencing grain prolamin content. For example, the expression of Tel_gamma_gliadin_1E_8 was about 5–6 times than that of Tel_gamma_gliadin_1E_2. Slight differences in gene expression were found in the α-gliadin, LMW-GS and HMW-GS gene subfamilies. In addition, different prolamin gene subfamilies had their own specific expression patterns. In α-gliadin and LMW-GS gene subfamilies, the expression level of these genes in the half-grain stage was higher than that in the grain stage, which indicated that expression of these genes increased at first and then decreased [27]. However, expression of γ-gliadin genes was increasing and we speculate that they will decline rapidly or slowly in the future due to reduced gene activity with the maturity of grains. In the HMW-GS gene subfamily, the expression of x-type HMW-GS genes was different from that of y-type. The expression of Tel_hmw_glutenin_1E_x in the half-grain stage was higher than that in the grain stage, while the expression of Tel_hmw_glutenin_1E_y increased slightly from the half-grain to the grain stage.

In addition, 59 starch synthesis related genes were found in *Th. elongatum* by homologous sequences search of previous reported wheat starch metabolic related genes [34]. Expression of these genes at six grain development stages was investigated and profiled (Additional file 5). Of the 4 expressed aldolases (ALDs), obvious pattern was observed that these genes were expressed before ovary expansion, whose function were produce fructose-6-P through the gluconeogenic pathway and then convert to glucose-6-P by PGIs to maintain carbon flux toward starch formation [34]. AGPL1 and AGPS1 were expressed at the early stage of grain development, which was consisted with previous study [35]. GBSSI, a granule-bound starch synthase enzyme, expressed at the beginning of grain development, then reached the peak at the half grain stage, suggesting an important role in the seed development [36]. Nevertheless, a large number of starch synthesis-related genes were not expressed in *Th. elongatum*, which may give a explanation why its seeds is much smaller than common wheat. Overall, although some starch metabolic related genes follow similar express pattern as gluten genes in *Th. elongatum*, but most of them showed different patterns, how genes in protein and starch metabolic pathways are coordinated for the gliadins and glutenins accumulation need more research.

### Electrophoretic maps and kneading quality performance of disomic substitution lines

**Fig. 6:**
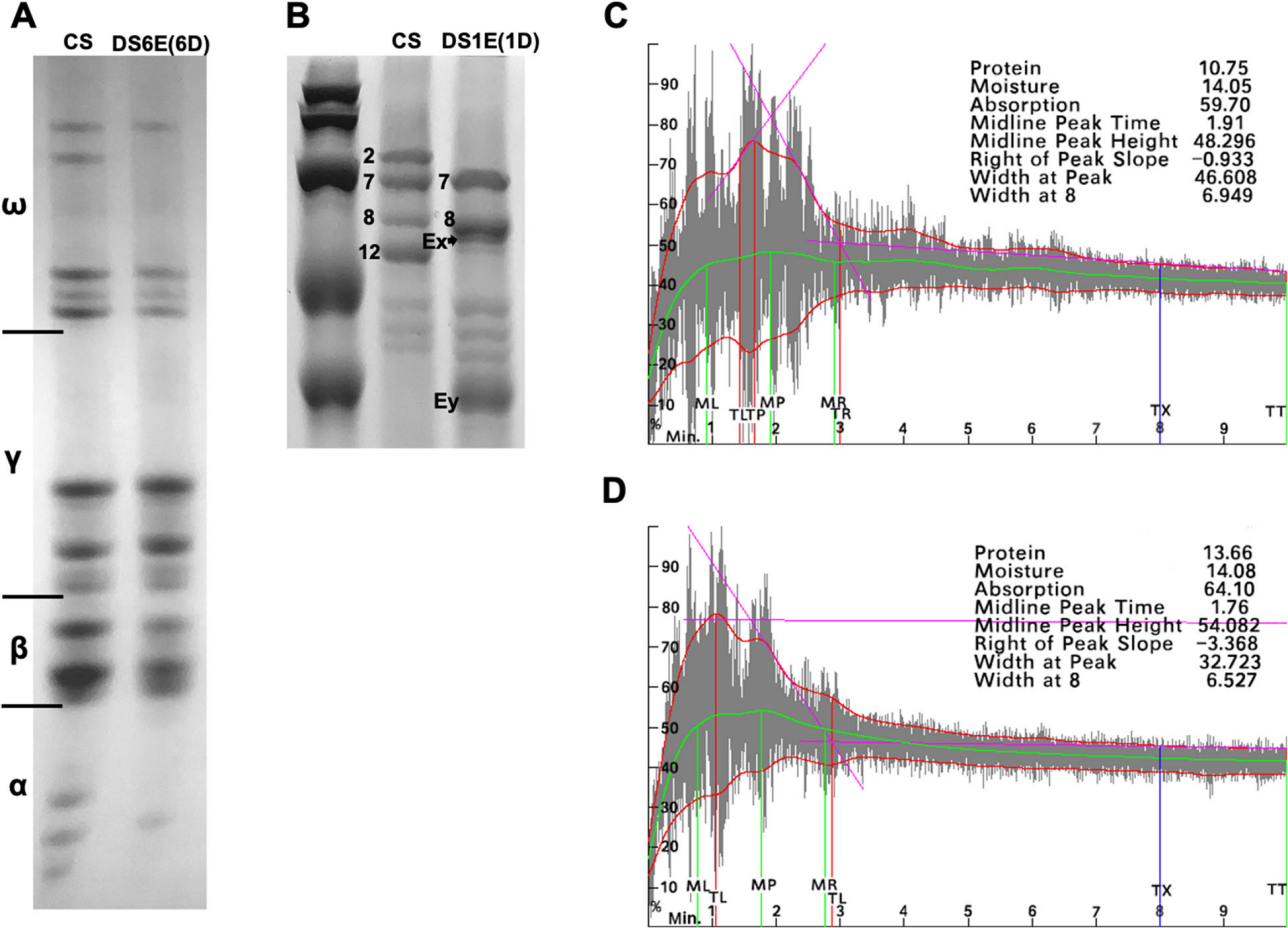
Electrophoretic map and rheological properties. **A** A-PAGE electrophoretic patterns of gliadins of CS and DS6E(6D). **B** 15% SDS-PAGE electrophoretic patterns of glutenins from CS, DS1E(1D). **C**-**D** Rheological properties of CS, DS1E(1D) respectively

In order to investigate the potential usage of *Th. elongatum* prolamin family in wheat high quality breeding, we have speculated the protein expression and processing quality of *T. aestivum*-*Th. elongatum* disomic substitution lines. Considering the distribution pattern of prolamin genes on chromosomes, the substitution lines of DS1E(1D) and DS6E(6D) were mainly studied, which have been identified by genetic markers and cytological approaches (Additional file 6) [37]. The results of A-PAGE electrophoretic map showed that most α-gliadins disappeared in the substitution line DS6E(6D) (Fig. 6**a**), and the two visible bands for β-gliadins became weakened. This result is consistent with the fact that less α-gliadins were expressed in *Th. elongatum* (Fig. 5)*,* indicating its potential application for quality breeding to reduce CD causal epitopes. Interestingly, we found one ω-gliadin band in wheat became silent in this substitution line, which may due to gene silencing during the process of chromosome engineering (Fig. 6**a**). Even a α-gliadin gene cluster have uniquely evolved on the chromosome 7E (Fig. 2), we found no significant difference in the A-PAGE map of DS7E(7D) compared with the control CS (Additional file 7), which was consistent with the transcriptome results that no genes in this cluster were abundantly expressed (Fig. 5).

HWM-GS accounts for 16% percent of the total wheat gluten protein, but the quality of wheat or gluten quality has a decisive role [38]. Consist with previous reports, CS contained the HMW-GSs of 7 + 8 subunits (from B subgenome), and 2 + 12 subunits (from D subgenome), named as Glu-B1 and Glu-D1, respectively (Fig. 6**b**). In the substitution line of DS1E(1D), 2 + 12 subunites of Glu-D1 loci were proposed to be substituted by the HWM-GS from E genome, both Ex and Ey subunits were detected in DS1E(1D) (Fig. 6**b**). Next, kneading parameters were speculated to check the kneading performance of DS1E(1D). The protein content of DS1E(1D) was 3% higher than that of CS (Fig. 6**c and d**). There was no significant difference in width at 8 min between CS and DS1E(1D), but the peak height of DS1E(1D) increased, indicating that the introduction of 1E chromosome enhanced the kneading resistance of dough (Fig. 6**c and d**). This is consistent with previous result that the HMW-GSs of E genome may be novel genetic resource for wheat dough quality improvement [28], and the short segment translocation lines can be further created to verify its breeding value.

## Discussion

Prolamin gene families play important roles in flour viscoelasticity, nutritional quality, and CD epitope content [14–16, 39]. Currently, knowledge is still limited about prolamin genes in *Th. elongatum*, a well-known species for wheat distant breeding. In this study, we identified 19 α-gliadins, 9 γ-gliadins, 19 ω-gliadins, 2 HMW-GSs, and 5 LMW-GSs in the *Th. elongatum* genome. Comparison of prolamin genes among four Triticeae species revealed dynamic changes regarding to gene numbers and pseudogene rates, which indicated the complexity and variable of this gene family.

Detailed synteny analysis showed that although the order of ω-gliadin, γ-gliadin and LMW-GS genes on the first homologous group was maintained among the four studied species, local rearrangement and copy number variation were observed in this interval, revealing a dynamic change in this region (Fig. 3). Similar results were also found in α-gliadins and HMW-GS subfamilies. Further classification of duplication genes showed that each gene subfamilies was largely composed with tandem duplications (73.7% for α-gliadins, 88.9% for γ-gliadins, 68.4% for ω-gliadin and 50% for LMW-GSs). The tandem duplication should have driven fast evolution for these prolamin genes.

Phylogenetic analysis of prolamin genes among four studied species showed that genes from the same species were tended to be clustered together. Besides, some genes were not well clustered according to species, which may due to incomplete or large changes in the protein sequences, especially HMW-GS and ω-gliadin genes from common wheat, which all have a large proportion of gap regions (Additional file 1: Table S3). In addition, many of these genes have been lost or newly evolved after speciation, possibly due to unequal cross-over or gene conversion (Fig. 4, Additional files 3 and 4) [40]. For instance, *Th. elongatum* has evolved 19 copies of ω-gliadin genes, while many copies in clade 2, clade 3 were apparently duplicated after diverging from the other species (Additional file 4). Similarly, many α-gliadin genes in clade 1 to clade 4 are likely lost and resulting in fewer α-gliadins in *Th. elongatum* genome (Fig. 4 and Fig. 6**a**). This fast evolution of prolamin family is accompanied with high rates of pseudogenes and large genetic diversity inner- or inter-species, and the richness of these seed-storage protein resources favor future wheat quality breeding.

According to previous reports of common wheat, the expression of LMW-GS genes began to express at the 5th day and reached the peak at the 10 ~ 14th day after anthesis, and then decreased with the maturity of seeds [27]. In *Th. elongatum*, the expression of three LMW-GS genes showed a consistent trend (Fig. 5). For the gliadin genes, their expression has genomic differences that genes from B and D subgenomes are early-expressed (highest level at 10 days after flowering), similar to the expression of LMW-GS genes, while those of the A genome are late-expressed genes (highest level at 20 days after flowering) [27]. The expression trend of α-gliadin genes is synchronous with that of LMW-GS genes in *Th. elongatum*, as early-expressed genes (Fig. 5). However, the expression of γ-gliadin genes increased from the half grain to the grain stage and reached its peak later than that of α-gliadin genes and LMW-GS genes, which demonstrated different roles of these prolamin genes in the grain development process.

Based on the putative functional α-gliadin sequences of four species, our results showed that CD peptides were genome-specific, which is consistent with the results of a previous study (Additional file 1: Table S5) [1]. Importantly, α-gliadins of the *Th. elongatum* genome contain only one type CD peptide, which is beneficial to low-CD breeding. Moreover, although α-gliadin genes were both found in the sixth homologous group of CS wheat and *Th. elongatum*, but the CD peptide of α-gliadins in *Th. elongatum* genome is much fewer than that in D genome (Additional file 1: Table S5). The grain of DS6E(6D), which replaces the chromosome 6D genome with the chromosome 6E genome and has been verified through cytological study (Additional file 6), were speculated for protein content by A-Page, and the protein bands for α-gliadin genes did largely disappeared (Fig. 6**a**). This result indicates that the α-gliadins locus on D genome can be further translocated by short E genome fragment by distant hybridization, generating less toxic wheat varieties for CD patient population. Moreover, gene silencing may also favor such utility during engineering alien chromatin into wheat background [41]. In DS6E(6D), we did found one band of ω-gliadin was silenced (Fig. 6**a**).

## Conclusions

Prolamins, the major protein component in cereal endosperm, play important roles for human diet. Here, we characterized and compared this gene family in *Th. elongatum* and three other wheat relatives*.* We explained the different expression pattern of prolamin gene subfamilies, found the dynamic changes of gene intervals, elaborated the evolutionary relationship among the Triticeae species used in this study. Our results showed that the HMW-GSs from *Th. elongatum* genome had highly potential for future improvement of wheat dough quality. Also, the *Th. elongatum* genome with low content of small CD peptides will be profitable genomic choice for the cultivation of wheat with low celiac disease.

## Methods

### Identification of prolamin genes

Genome datasets and annotation files of four species (Th. elongatum, T. aestivum, T. urartu, and Ae. tauschii) were downloaded from the Genome Warehouse (https://bigd.big.ac.cn/gwh/Assembly/965/show), IWGSC (https://urgi.versailles.inra.fr/download/iwgsc/IWGSC_RefSeq_Annotations/v1.1/), MBKBASE (http://mbkbase.org/Tu/), and http:// aegilops.wheat.ucdavis.edu/ATGSP/annotation/, respectively. Coding sequences and protein sequences of prolamin genes in T. aestivum, T. urartu, and Ae. tauschii were extracted from corresponding files [14–16]. The common wheat prolamin gene sequences, including HMW-GS, LMW-GS, α-, δ-, γ-, and ω-gliadin genes, were used as queries in blastn searches against the Th. elongatum genome sequence with an e-value cutoff of 1 × 10^− 10^, and then matched genes with full-length sequences (including stop codon) were extracted and manually annotated [16]. All CDSs and protein sequences were regenerated using the gffread program [42].

### Feature analysis

Putative functional genes were deduced according to whether the amino acid sequences of prolamins could produce proteins with complete structure. The pseudogene rate of the gene family was calculated with the number of pseudogenes divided by the total number of genes. Two genes were deleted because the sequences were not completed. All putative functional prolamin sequences were submitted to Expasy (https://web.expasy.org/compute_pi/) for calculation of molecular weight (MW) and theoretical isoelectric point (pI).

### Distribution and duplication of prolamin genes

The distribution of prolamin genes on chromosomes was determined by the MapChart software [43]. Three roles were used to determine whether they were tandem repeat genes [44, 45]: (a) The distance between two genes on the same chromosomal fragment was less than 100 kb; (b) the shorter aligned sequence covered > 70% of the longer sequence; (c) the similarity of aligned sequences was > 70%. If AB and BC are two pairs of tandem repeat genes (A, B, and C are three adjacent genes) but AC does not meet the tandem replication criteria, A and C are considered to be tandem repeat genes. The micro-synteny analysis was conducted using the JCVI software with appropriate parameters (cscore = 0.8) (https://github.com/tanghaibao/jcvi/wiki).

### Phylogenetic analysis and classification of each prolamin subfamily

Phylogenetic trees were constructed using CDSs of the conservative domains derived from α-gliadin, γ-gliadin, LMW-GS, and HMW-GS genes. The evolutionary tree of ω-gliadins was constructed using gene conservative sequences. Multiple sequence alignments were first performed using the MUSCLE program with default parameters [46]. Phylogenetic trees were constructed using the MEGA X software with the maximum likelihood method [47]. Bootstrap was set as 1000 replicates. Based on model tests, the best models of these phylogenetic trees were set to be TN93 + G, K2 + G, K2 + I, and T92 + I, K2 + G respectively [48].

### CD content detection

The key peptides involved in the pathogenesis of CD are DQ2.5-glia-α1a (PFPQPQLPY), DQ2.5-glia-α1b (PYPQPQLPY), DQ2.5-glia-α2 (PQPQLPYPQ), DQ2.5-glia-α3 (FRPQQPYPQ), and a 33-mer peptide (LQLQPFPQPQLPYPQPQLPYPQPQLPYPQPQPF). These small peptide sequences were retrieved from the deduced functional protein sequences, and the contents of these small peptides of single prolamin subfamily in the genomes of the four species were determined.

### Transcriptome analysis

In order to analyze the expression patterns of prolamin genes in *Th. elongatum*, we sampled tissues at six stages with three biological replicates: young spike, pre-flowering, flowering, ovary expansion, half-grain, and grain stages. Total RNA was extracted for each sample using TRIzol® Reagent (Invitrogen) according to the manufacturer’s instructions. A total of 18 samples were paired-end sequenced on the Illumina HiSeq 4000 platform. The quality of all samples was checked by fastqc (v0.11.9) and controlled by fastp (v0.20.0) with default parameters [49, 50]. Hisat2 (v2.2.1) was used to map clean reads to the genome of *Th. elongatum* [51]. The transcripts per kilobase of exon model per million mapped reads (TPM) value for each gene was calculated by StringTie (v2.1.2) and the heatmap was constructed by TBtools (v1.047) [52, 53].

### Protein extraction and gel electrophoresis

#### The method for glutenins

Single milled seed was suspended in 0.5 mL mixture extraction liquid and then incubated at 65 °C for 2 h. The components of the extracted liquid mixture were as follows: 25 mL Tris-HCl (0.5 M, pH 6.8), 20 mL glycerol, 4 g sodium dodecyl sulfate (SDS), 30 g bromophenol blue, and 1 g dithiothreitol (DTT). The total volume of above extracted liquid mixture was then made to 100 mL with deionized water. The glutenin subunits were separated by a discontinuous sodium dodecyl sulfate polyacrylamide gel electrophoresis (SDS-PAGE) system. The 100 mL 15% separating gel included 25 mL deionized water, 50 mL 30% Acryl/Bis solution (29:1), 25 mL 4X separation gel buffer, 1 mL 10% (m/v) ammonium persulfate solution and 800 uL TEMED. The 80 mL 5% stacking gel included 45.6 ml deionized water, 13.6 mL 30% Acryl/Bis solution (29:1), 20 mL concentrated rubber buffer, 800 uL 10% (m/v) ammonium persulfate solution and 65 uL TEMED. Gels were run at a constant current (12 mA) for 20 h.

#### The method for gliadins

Single milled seed was suspended in 0.3 mL 70% ethanol and then incubated at 37 °C for 2 h. Acid gel composition was as follows: 0.5 g ascorbic acid, 0.01 g FeSO_4_, 50 g acrylamide, and 2.5 g Bis-Tris. The total volume of each sample was then made to 500 mL with deionized water. The separating gel included 40 mL acid gel liquid, 0.3 mL formic acid, and 30 uL 0.6% H_2_O_2_. The stacking gel included 10 mL acid gel liquid and 0.1 uL 0.6% H_2_O_2_. Gels were run at a constant voltage in two stages (150 V for 30 min, then 350 V for 4 h).

Coomassie brilliant blue dye liquor included 1 g coomassie brilliant blue R-250, 100 mL glacial acetic acid, 250 mL isopropanol and 650 mL deionized water. The decolorizing solution included 50 mL anhydrous ethanol, 100 mL glacial acetic acid and 850 mL deionized water. Both gels were stained with a Coomassie brilliant blue dye liquor for 4 h and destained overnight.

#### Fish

FISH was performed with the probes oligo-psc119.2–1 from *Secale cereal* and oligo-pta535–1 from *T. aestivum*. Hybridization solution was prepared according to the number of samples prepared. The two probes were mixed at a ratio of 1:1 before hybridization. The specific methods used were performed according to previous method [54]**.**.

#### Dough rheological properties test

Mature grains were milled into flour using a mill for further testing. In this study, the protein and water contents of flour were determined by a DA7200 multi-function near infrared analyzer. By referring to the formula of the “AACC54-40A” method, the main mixing parameters, such as the mixing time, middle peak height, middle peak time, middle peak at 8 min, and width at 8 min, were determined with a 10 g mixograph.

## Supplementary Information

**Additional file 1: Table S1.** Prolamin genes in *Th. elongatum* genome. **Table S2.** Error correction of prolamin gene annotations in *T. aestivum*, *Ae. tauschii* and *T. urartu*. **Table S3.** Coding sequences for the prolamin genes in the four studied species. **Table S4.** Molecular characteristics of prolamin genes. **Table S5.** The distribution of CD epitopes in the genomes of *T. aestivum*, *T. urartu*, *Ae. tauchii* and *Th. elongatum*. **Table S6.** Transcriptome data of *Th. elongatum* at different development stages.

**Additional file 2:** Mirco-synteny analysis of each prolamin gene family between *Th. elongatum* and three related species. Red lines highlight the syntenic prolamin gene pairs and gray lines indicate the other syntenic gene pairs. (A) Synteny relationship of α-gliadins. (B) Synteny relationship of HMW-glutenins.

**Additional file 3:** Phylogenetic tree for each prolamin subfamilies. (A) Phylogenetic tree of γ-gliadin family. *X13508.1* was set as outgroup. (B) Phylogenetic tree of LMW-GS gene family. *HQ293220.1* was set as outgroup. (C) Phylogenetic tree of HMW-GS gene family. *FJ481574.2* was set as outgroup.

**Additional file 4:** Phylogenetic tree of ω-gliadin gene family. *FJ600500* was set as outgroup.

**Additional file 5:** Expression profile of 59 starch synthesis related genes in *Th. elongatum*. Genes were named according to names in common wheat.

**Additional file 6:** The FISH pattern of DS1E(1D) and DS6E(6D). Chromosomes 1E and 6E from *Th. elongatum* were indicated by arrows respectively.

**Additional file 7:** A-PAGE patterns of gliadin in CS and its derived substitution lines.

## Data Availability

The transcriptome data used in this study have been uploaded to NCBI under BioProjectID PRJNA540081 (https://www.ncbi.nlm.nih.gov/sra/PRJNA540081). The other data used to support the findings of this study are available from the corresponding author upon request.

## Abbreviations

HMW-GS: High-molecular-weight glutenin subunit
LMW-GS: Low-molecular-weight glutenin subunit
DS: Disomic substitution
CD: Celiac disease
CS: Chinese Spring
FISH: Fluorescence in situ hybridization
bp: Base pair
kb: Kilobase
pI: Isoelectric point
MW: Molecular Weight
SDS-PAGE: Sodium dodecyl sulfate polyacrylamide gel electrophoresis
A-PAGE: Acid polyacrylamide gel electrophoresis
