# Supplementary figures and images for: Genome-wide identification, characteristics and expression of the prolamin genes in Thinopyrum elongatum
Source: BMC Genomics. 2021 Dec 2;22:864. doi: 10.1186/s12864-021-08088-x (PMC8638145; doi:10.1186/s12864-021-08088-x)

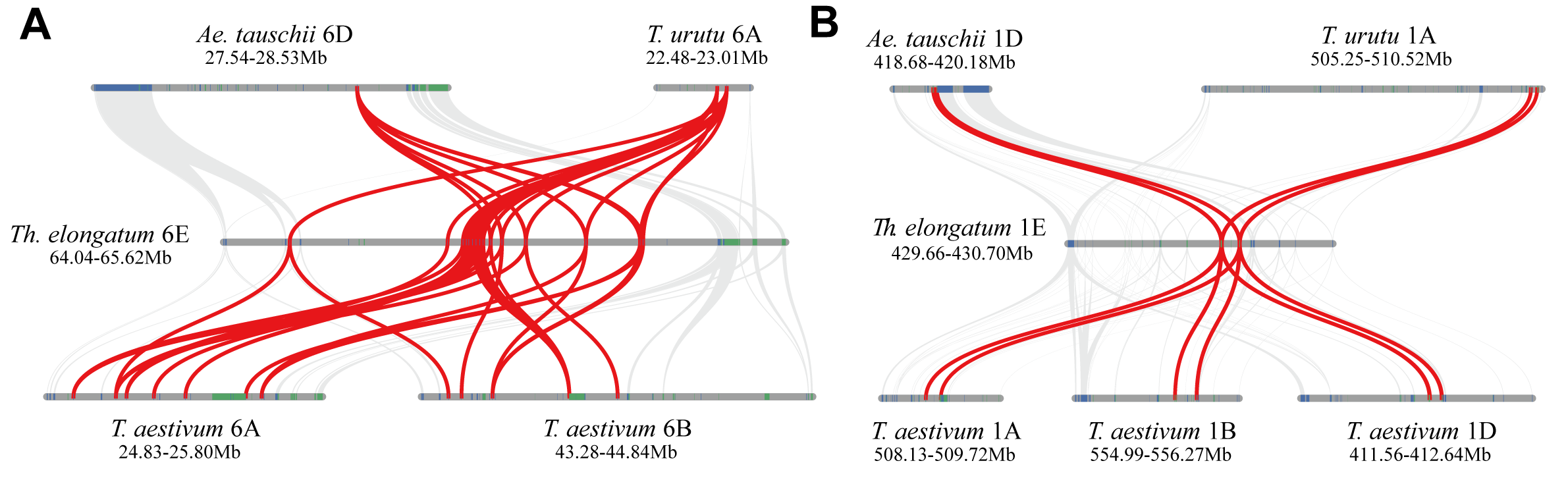

Supplement: Supplementary file 2 — Additional file 2: Mirco-synteny analysis of each prolamin gene family between Th. elongatum and three related species. Red lines highlight the syntenic prolamin gene pairs and gray lines indicate the other syntenic gene pairs. (A) Synteny relationship of α-gliadins. (B) Synteny relationship of HMW-glutenins. [file 12864_2021_8088_MOESM2_ESM.tif]

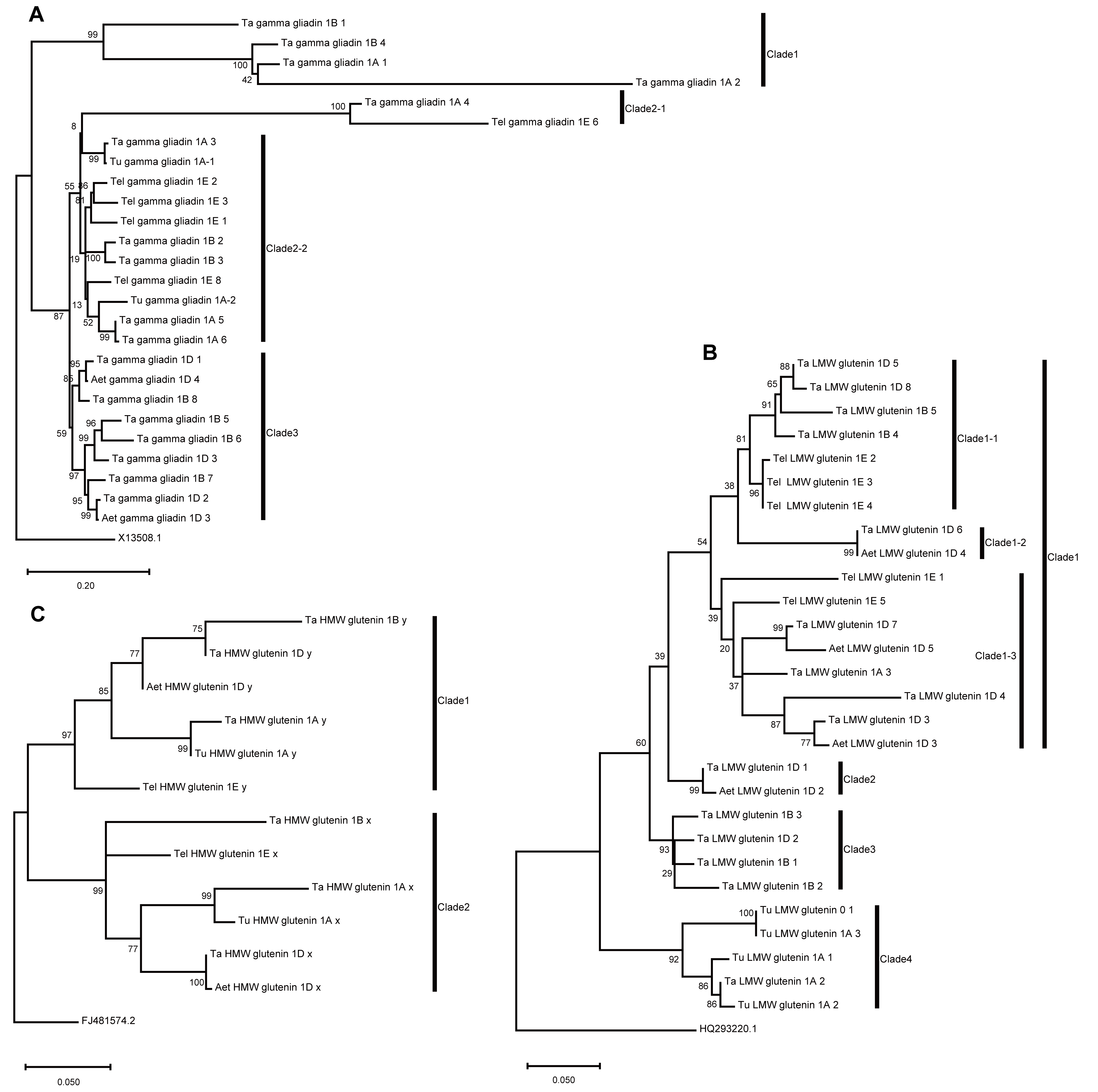

Supplement: Supplementary file 3 — Additional file 3: Phylogenetic tree for each prolamin subfamilies. (A) Phylogenetic tree of γ-gliadin family. X13508.1 was set as outgroup. (B) Phylogenetic tree of LMW-GS gene family. HQ293220.1 was set as outgroup. (C) Phylogenetic tree of HMW-GS gene family. FJ481574.2 was set as outgroup. [file 12864_2021_8088_MOESM3_ESM.tif]

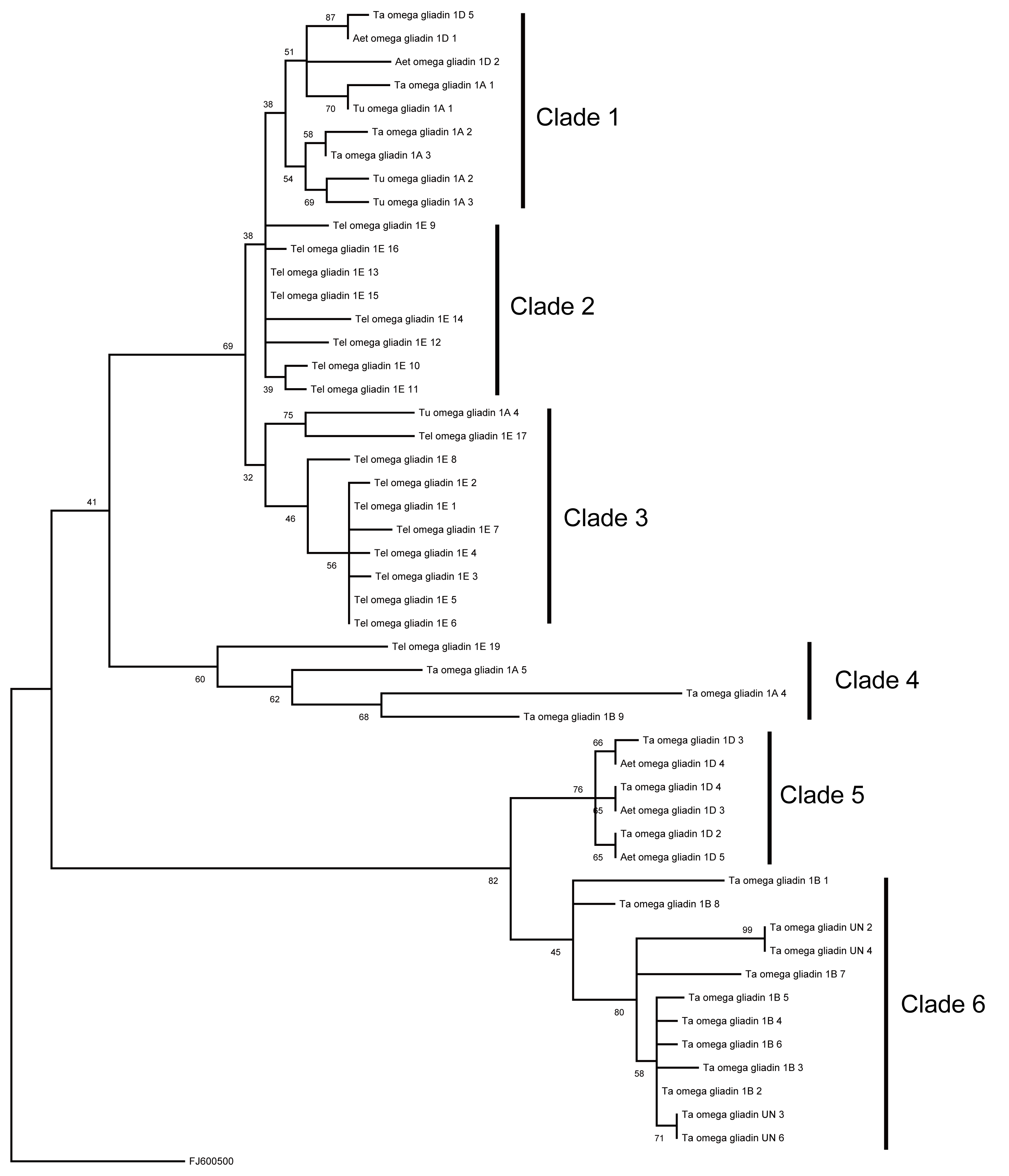

Supplement: Supplementary file 4 — Additional file 4: Phylogenetic tree of ω-gliadin gene family. FJ600500 was set as outgroup. [file 12864_2021_8088_MOESM4_ESM.tif]

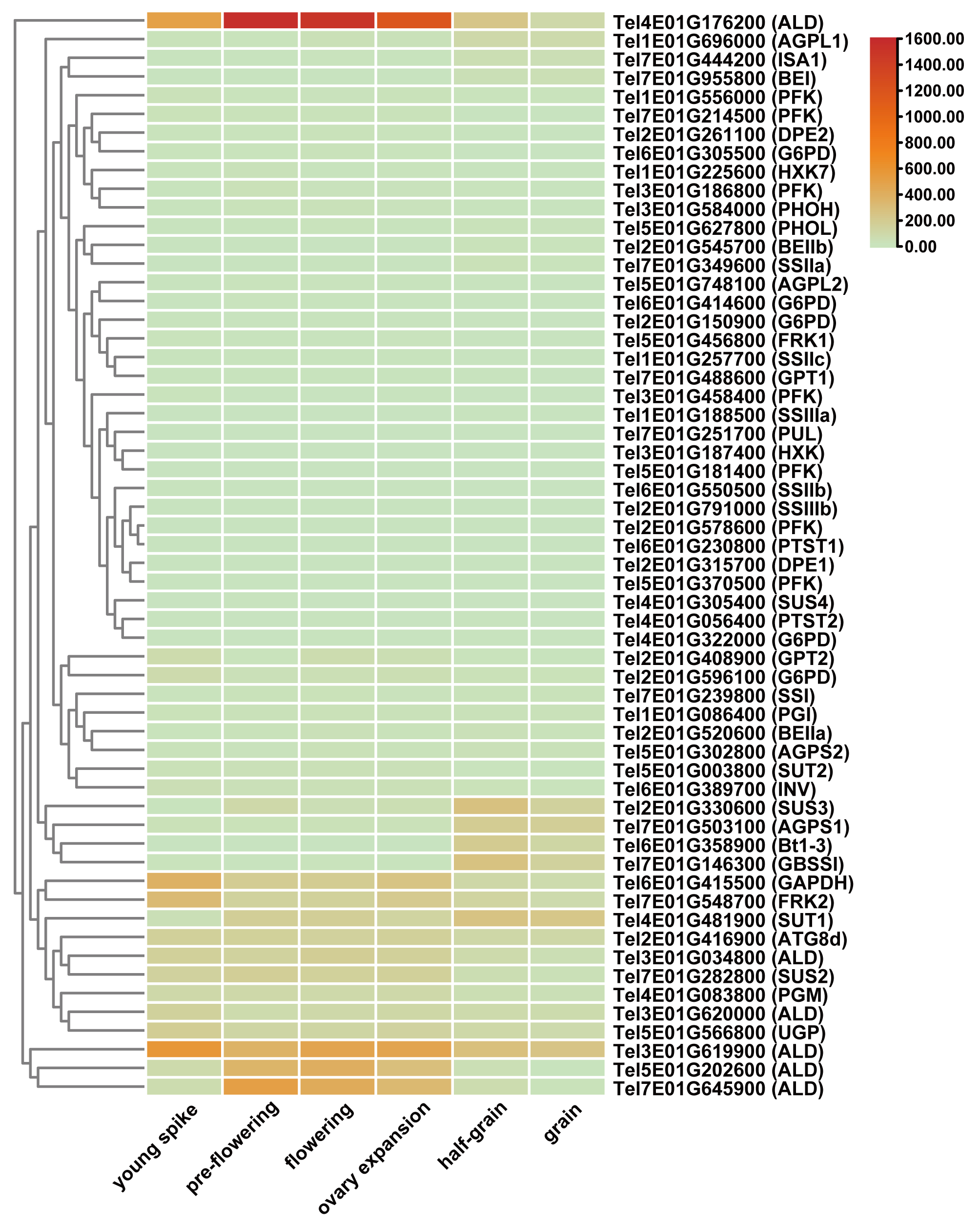

Supplement: Supplementary file 5 — Additional file 5: Expression profile of 59 starch synthesis related genes in Th. elongatum. Genes were named according to names in common wheat. [file 12864_2021_8088_MOESM5_ESM.tif]

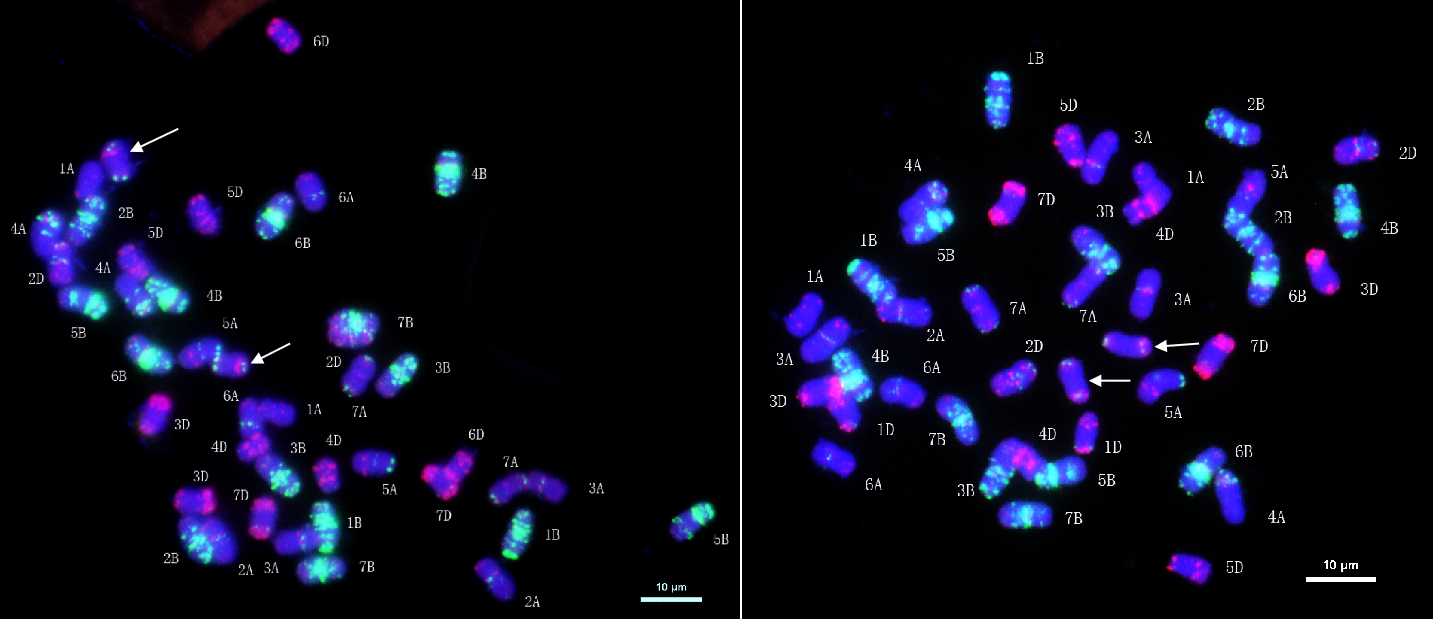

Supplement: Supplementary file 6 — Additional file 6: The FISH pattern of DS1E(1D) and DS6E(6D). Chromosomes 1E and 6E from Th. elongatum were indicated by arrows respectively. [file 12864_2021_8088_MOESM6_ESM.tif]

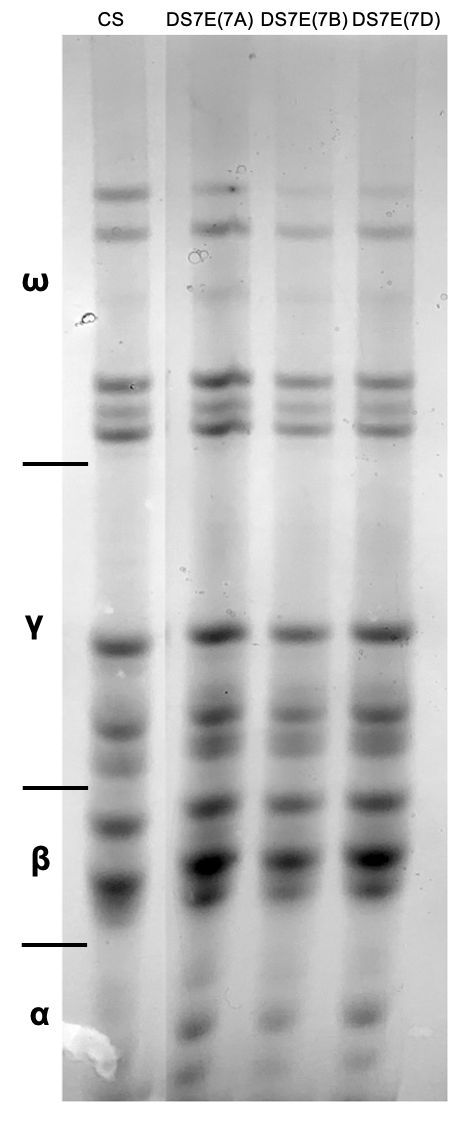

Supplement: Supplementary file 7 — Additional file 7: A-PAGE patterns of gliadin in CS and its derived substitution lines. [file 12864_2021_8088_MOESM7_ESM.tif]
